# The correlation study on chest CT features and kidney injury in severe COVID-19 pneumonia from a multicenter cohort study

**DOI:** 10.1007/s42058-022-00098-2

**Published:** 2022-06-02

**Authors:** Guan Li, Zhiyuan Sun, Song Luo, Lianli Qiu, Longjiang Zhang, Guangming Lu

**Affiliations:** Department of Medical Imaging, Jinling Hospital, Medical School of Nanjing University, No. 305, Eastern Zhongshan Road, Nanjing, 210002 China

**Keywords:** Coronavirus Disease 2019 (COVID-19), Estimated glomerular filtration rate (eGFR), Chest computed tomography, Crazy-paving pattern, Clinical prognosis

## Abstract

**Background:**

Among confirmed severe COVID-19 patients, although the serum creatinine level is normal, they also have developed kidney injury. Early detection of kidney injury can guide doctors to choose drugs reasonably. Study found that COVID-19 have some special chest CT features. The study aimed to explore which chest CT features are more likely appear in severe COVID-19 and the relationship between related (special) chest CT features and kidney injury or clinical prognosis.

**Methods:**

In this retrospective study, 162 patients of severe COVID-19 from 13 medical centers in China were enrolled and divided into three groups according to the estimated glomerular filtration rate (eGFR) level: Group A (eGFR < 60 ml/min/1.73 m^2^), Group B (60 ml/min/1.73 m^2^ ≤ eGFR < 90 ml/min/1.73 m^2^), and Group C (eGFR ≥ 90 ml/min/1.73 m^2^). The demographics, clinical features, auxiliary examination, and clinical prognosis were collected and compared. The chest CT features and eGFR were assessed using univariate and multivariate Cox regression. The influence of chest CT features on eGFR and clinical prognosis were calculated using the Cox proportional hazards regression model.

**Results:**

Demographic and clinical features showed significant differences in age, hypertension, and fatigue among the Group A, Group B, and Group C (all *P* < 0.05). Auxiliary examination results revealed that leukocyte count, platelet count, C-reactive protein, aspartate aminotransferase, creatine kinase, respiratory rate ≥ 30 breaths/min, and CT images rapid progression (>50%) within 24–48 h among the three groups were significantly different (all *P* < 0.05). Compared to Group C (all *P* < 0.017), Groups A and B were more likely to show crazy-paving pattern. Logistic regression analysis indicated that eGFR was an independent risk factor of the appearance of crazy-paving pattern. The eGFR and crazy-paving pattern have a mutually reinforcing relationship, and eGFR (HR = 0.549, 95% CI = 0.331–0.909, *P* = 0.020) and crazy-paving pattern (HR = 2.996, 95% CI = 1.010–8.714, *P* = 0.048) were independent risk factors of mortality. The mortality of severe COVID-19 with the appearance of crazy-paving pattern on chest CT was significantly higher than that of the patients without its appearance (all* P* < 0.05).

**Conclusions:**

The crazy-paving pattern is more likely to appear in the chest CT of patients with severe COVID-19. In severe COVID-19, the appearance of the crazy-paving pattern on chest CT indicates the occurrence of kidney injury and proneness to death. The crazy-paving pattern can be used by doctors as an early warning indicator and a guidance of reasonable drug selection.

## Introduction

From December 2019, the first case of unknown viral pneumonia was found in Wuhan, China. The World Health Organization (WHO) has officially named the unknown viral pneumonia Coronavirus Disease 2019 (COVID-19) pneumonia on 11 February 2020 [1]. To date, WHO has reported 214,824,267 confirmed cases and 4,474,048 cases deaths in the world [2]. Severe COVID-19 patients could rapidly progress to organ dysfunction, such as acute respiratory distress syndrome, acute cardiac injury and acute kidney injury and so on [3, 4]. According to the diagnosis and treatment program of COVID-19 (Trial Seventh Edition) issued by the National Health Commission of the People’s Republic of China, the clinical classification of COVID-19 includes mild, moderate, severe and critical types [5]. Severe COVID-19 needs to meet any of the followings: (I) severe respiratory distress (respiratory rate (RR) ≥ 30 breaths/min); (II) SpO_2_ < 93% at rest; (III) PaO_2_/FiO_2_ ≤ 300 mmHg. In addition, mild patient with lesion progression of more than 50% within 24–48 h should be treated as severe type.

Related studies have reported that COVID-19 infection can cause kidney injury [4, 6]. Among some patients with severe COVID-19, although the serum creatinine value remains normal, estimated glomerular filtration rate (eGFR) value turns abnormal. The previous studies have shown that eGFR can evaluate kidney injury more accurately than serum creatinine [7, 8]. According to the National Kidney Foundation Kidney Foundation Kidney Disease Outcomes Quality Initiative (NKF-K/DOQI), it is proposed that eGFR (mL/min/1.73 m^2^) ≥ 90 represents kidney injury with normal or raised; 60 ≤ eGFR (mL/min/1.73 m^2^) < 90 represents kidney injury with mild; eGFR (mL/min/1.73 m^2^) < 60 represents kidney injury with moderate or severe. Therefore, eGFR (mL/min/1.73 m^2^) < 90 can be used as an early effective index to evaluate kidney injury.

Various studies have reported that COVID-19 have certain chest CT features [9, 10]. Li et al. reported that the common chest CT features included ground-glass opacity (GGO), linear opacities, consolidation, interlobular septal thickening and crazy-paving pattern [11]. The crazy-paving pattern can be defined as diffuse or scattered ground-glass attenuation superimposed on a network of interlobular septal thickening and intralobular lines [12]. Johkoh et al. proposed that crazy-paving pattern represented an increase in the severity of the pathologic process at the borders of unit structures [13]. The purpose of our study was to determine what kinds of chest CT features are more likely appear in severe COVID-19 and what kinds of chest CT features may lead to kidney injury and poor clinical prognosis in severe COVID-19.

## Methods

### Patient population

Patients (*n* = 162; 105 males and 57 females, median age 55.7 ± 14.8 years, range from 21 to 91 years) with severe COVID-19 patients admitted to 13 medical centers in China from January 15, 2020 to February 20, 2020 were reviewed. According to the level of eGFR, 162 patients were divided into three groups: Group A (eGFR < 60 ml/min/1.73 m^2^), Group B (60 ml/min/1.73 m^2^ ≤ eGFR < 90 ml/min/1.73 m^2^), and Group C (eGFR ≥ 90 ml/min/1.73 m^2^). This study, performed following the Declaration of Helsinki, has been approved by all of the institutional review boards.

All patients’ clinical data, laboratory data and chest CT images were collected and reviewed by two radiologists with 20 and 10 years of experience (Z.Y. S. and G. L.). All baseline data collected on the first day in-hospital included: age, sex, contact history (travel or residence history in Wuhan and the local community with confirmed patient), respiratory rate (RR), fever, cough, myalgia, fatigue, headache, nausea, diarrhoea, abdominal pain, dyspnea, comorbidities (cardiovascular disease, diabetes, hypertension, chronic obstructive pulmonary disease (COPD), chronic liver disease, chronic kidney disease and malignancy). The baseline laboratory data included: leukocyte count (LC), platelet count (PC), C-reactive protein (CRP), aspartate aminotransferase (AST), creatine kinase (CK) and lactate dehydrogenase (LDH). The Chronic Kidney Disease Epidemiology Collaboration (CKD-EPI) creatinine equation was used to calculate the eGFR [14]. The blood gas analysis included: SpO_2_, PaO_2_ and FiO_2_. Patients with rapid progression (>50%) of Chest CT features and CT images within 24–48 h were collected.

### Inclusion and exclusion criteria

The inclusion criteria were as follows: (I) clinically confirmed COVID-19 (COVID-19 nucleic acid or gene sequence (+)); (II) confirmed severe COVID-19 (RR ≥ 30 breaths/min; SpO_2_ < 93% at rest; PaO_2_/FiO_2_ ≤ 300 mmHg; and rapid progression (>50%) on CT images within 24–48 h); (III) underwent serum creatinine and chest CT examination.

The exclusion criteria were as follows: (I) patients with renal dysfunction before COVID-19 infection; (II) pregnant women or children; (III) incomplete clinical data, laboratorial data and chest CT images; (IV) significant artifacts on chest CT images affected diagnosis.

### Chest CT image acquisition

All patients adopted supine position and held breath. The scanning ranged from the apex to the bottom of the lung. A Siemens Emotion 16 scanner CT (Siemens Healthineers; Erlangen, Germany) was applied to scan 18 patients from Yichang (Yichang Central People's Hospital, Yichang, Hubei, China) or Wuhan (General Hospital of the Yangtze River Shipping, Wuhan, Hubei, China) with a layer thickness of 5 mm. A GE Discovery CT750 HD (GE Healthcare, Milwaukee, Wis, USA) was adopted to scan 46 patients from Wenzhou (Wenzhou Central Hospital, Wenzhou, Zhejiang, China), Xiaogan (Xiaogan Central Hospital of Wuhan University of Science and Technology, Xiaogan, Hubei, China) or Wuhan (Wuhan No.1 Hospital, Wuhan, Hubei, China) with a layer thickness of 5 mm. A Siemens second generation 64-slice dual-source CT scanner (SOMATOM Definition Flash, Siemens Healthcare, Erlangen, Germany) was used to scan 40 patients from Shenyang (The First Hospital of China Medical University, Shenyang, Liaoning, China), Huangshi (Huangshi Central Hospital, Huangshi, Hubei, China) or Shiyan (Taihe Hospital, Hubei University of Medicine, Shiyan, Hubei, China) with a layer thickness of 5 mm. A Philips Ingenuity core 128 spiral CT scanner (Philips Medical Systems, Best, the Netherlands) was used to scan 17 patients from Xiangyang (Xiangyang Central Hospital, Xiangyang, Hubei, China) or Xuzhou (The Affiliated Hospital of Xuzhou Medical University, Xuzhou, Jiangsu, China) with a layer thickness of 1.5 mm. A Siemens Emotion 16 VC20B 16-slice spiral CT scanner (Siemens Healthcare GmbH, Erlangen, Germany) was used to scan 27 patients from Huanggang (Huanggang Central Hospital, Huanggang, Hubei, China) or Jingzhou (Jingzhou Central Hospital, The Second Clinical Medical College, Yangtze University, Jingzhou, Hubei, China), using 1.5 mm slice thickness. A NeuViz 64 In CT scanner (Neusoft Medical System, China) was used to scan 14 patients from Hainan (Hainan General Hospital, Haikou, Hainan, China) with a layer thickness of 5 mm. All chest CT images were non-contrast-enhanced CT scan.

### Chest CT characteristic

**Fig. 1:**
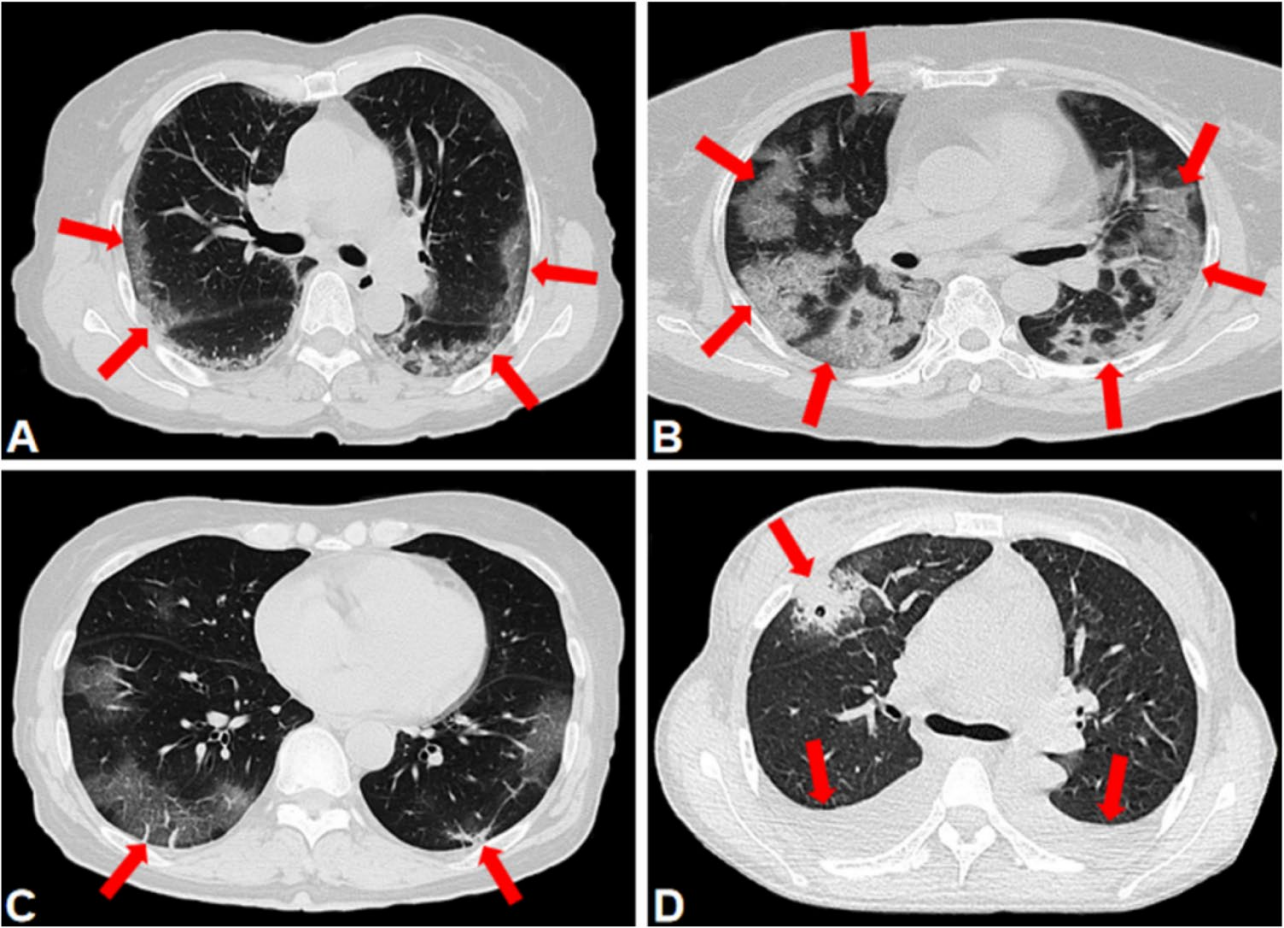
Chest CT features of COVID-19. **A** Male, 65 years, confirmed as COVID-19, chest CT images showed crazy-paving pattern and interstitial change (arrows); **B** Male, 74 years, confirmed as COVID-19, chest CT images showed GGO with consolidation, crazy-paving pattern, multiple, and irregular shape (arrows); **C** Male, 68 years, confirmed as COVID-19, chest CT images showed GGO and interstitial change (arrows); **D** Male, 59 years, confirmed as COVID-19, chest CT images showed GGO with consolidation and pleural effusion (arrows)

**Fig. 2:**
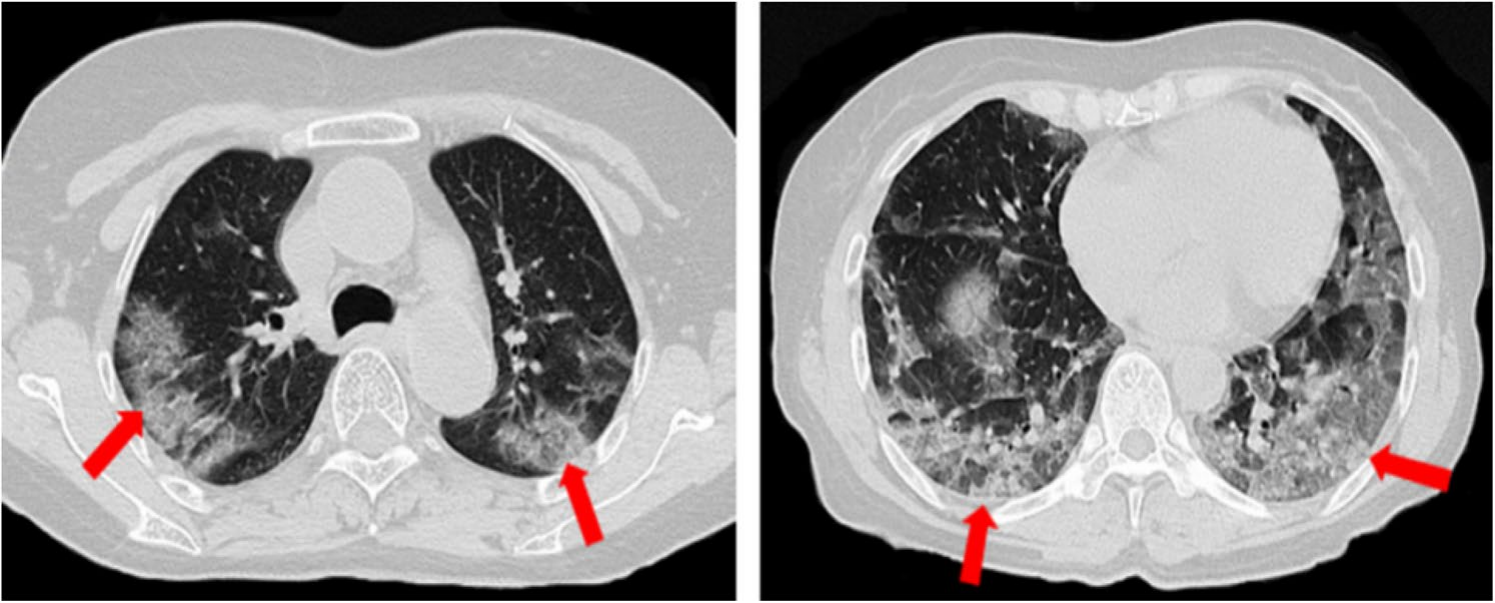
Chest CT feature the crazy-paving pattern. Male, 68 years, confirmed as COVID-19, chest CT images showed crazy-paving pattern (arrow)

According to the peer-reviewed literature on COVID-19 chest CT characteristic and the Fleischner Society glossary of terms [3], the chest CT features were summarized as follows: (I) the number of lesions (single or multiple); (II) the number of lesion-involved lung segment (0 ≤ numbers ≤ 20); (III) the shape of lesions (round or irregular shape); (IV) the density of lesions (GGO, consolidation, GGO with consolidation); (V) the crazy-paving pattern (GGO with superimposed interlobular and intralobular septal thickening); (VI) the interstitial changes; and (VII) the pleural effusion (Figs. 1, 2).

### Clinical prognosis

The impact of special chest CT features on clinical prognosis were also analyzed. We selected the mortality (No.), the number of patients entering intensive care unit (ICU) (No.), and mechanical ventilation adoption (No.) to evaluate the clinical prognosis.

### Statistical analysis

SPSS version 17.0 (SPSS Inc. Chicago, IL, USA) was used for statistical analysis. Normal distribution data were shown as mean ± SD; non-normal distribution data were shown as median (interquartile range) [M(IQR)]. Qualitative data were expressed as the number of cases and the percentage [*n*(%)]. Qualitative data were analyzed using the chi-square (*χ*^2^) or Fisher’s exact test. Normal distribution variables were analyzed using the Student’s *t*-test. Bonferroni’s adjustment was used to correct for multiple testing. Logistic regression analysis was used to estimate the significant variables (*P* < 0.05). Receiver operating characteristic (ROC) curve analysis was performed to determine the cut-off value.

Clinical prognosis was assessed using Kaplan–Meier survival analysis, and the influence of eGFR and chest CT features on clinical outcomes were calculated using the Cox proportional hazards regression model. Multivariate Cox regression analysis was used to determine the independent predictors of clinical prognosis. A *P* value < 0.05 was statistically significant.

## Results

### Demographic and clinical features

Demographic and clinical features between Group A, Group B and Group C are shown in Table 1. Age, hypertension and fatigue among the three groups have a significant difference (all *P* < 0.05). the intergroup comparisons showed that the age was older in Group A than that in Group C (63.2 ± 17.0 vs. 53.1 ± 12.9, *P* < 0.017), the hypertension in Group A higher than that in Group C (65% vs. 33%, *P* < 0.017), and the fatigue in Group B was less than that in Group C (14% vs. 47%, *P* < 0.017). The leukocyte count in group A was significantly more than that in group B (8.2 ± 4.1 vs. 5.3 ± 2.0, *P* < 0.017). The PC in Groups A and B were significantly less than that in Group C. The CPR, AST, CK, RR ≥ 30 breaths/min, and CT images rapid progression (>50%) within 24–48 h between Group A and Group C were significantly different (all *P* < 0.017).

**Table 1:** Demographic and clinical features of Groups A, B and C

Variable	Group A (*n* = 26)	Group B (*n* = 37)	Group C (*n* = 99)
Demographics			
Age, mean ± SD (years)	63.2 ± 17.0^*^	56.8 ± 16.1	53.1 ± 12.9
Sex, No. (%)			
Male	20 (77)	22 (59)	63 (64)
Female	6 (23)	15 (41)	36 (36)
Contact history, No. (%)			
Yes	14 (54)	19 (51)	51 (52)
No	12 (46)	18 (49)	48 (48)
Underlying diseases			
Cardiovascular disease, No. (%)	7 (27)	5 (14)	18 (18)
Diabetes, No. (%)	7 (27)	5 (14)	17 (17)
Hypertension, No. (%)	17 (65)^*^	18 (49)	33 (33)
COPD, No. (%)	2 (8)	3 (8)	4 (0)
Chronic liver disease, No. (%)	1 (4)	0 (0)	5 (5)
Chronic kidney disease, No. (%)	0 (0)	0 (0)	0 (0)
Malignancy, No. (%)	0 (0)	4 (11)	7 (7)
Signs and symptoms			
Fever, No. (%)	22 (85)	33 (89)	86 (87)
Cough, No. (%)	16 (62)	22 (59)	76 (77)
Myalgia, No. (%)	5 (19)	6 (16)	16 (16)
Fatigue, No. (%)	9 (35)	5 (14)^†^	47 (47)
Headache, No. (%)	2 (8)	5 (14)	13 (13)
Nausea, No. (%)	4 (16)	2 (5)	14 (14)
Diarrhoea, No. (%)	3 (12)	4 (11)	17 (17)
Abdominal pain, No. (%)	1 (4)	0 (0)	5 (5)
Dyspnea, No. (%)	13 (50)	13 (35)	43 (43)
Auxiliary examination			
LC, ×10^9^/L	8.2 ± 4.1^#^	5.3 ± 2.0	6.6 ± 3.7
PC, ×10^9^/L	146.9 ± 69.2^*^	153.4 ± 65.2^†^	187.4 ± 75.9
CRP, mg/L	72.4 ± 79.0^*^	53.0 ± 51.1	39.0 ± 35.9
AST, U/L	81.2 ± 48.5^*^	55.7 ± 33.3	39.4 ± 36.8
CK, U/L	629.8 ± 681.9^*^	320.7 ± 497.0	184.4 ± 243.0
LDH, U/L	410.1 ± 215.6	355.6 ± 170.8	340.1 ± 157.8
PaO_2_/FiO_2_ ≤ 300 mmHg, No. (%)	21 (81)	28 (76)	78 (79)
RR ≥ 30 breaths/min, No. (%)	17 (65)^*^	17 (46)	36 (36)
SpO_2_ < 93% at rest, No. (%)	19 (73)	26 (70)	72 (73)
CT images rapid progression (>50%) within 24–48 h, No. (%)	9 (35)^*^	5 (14)	13 (13)

*LC* leukocyte count, *PC* platelet count, *eGFR* estimated glomerular filtration rate, *CRP* c-reactive protein, *AST* aspartate aminotransferase, *CK* creatine kinase, *LDH* Lactate dehydrogenase, *RR* respiratory rate

*P* value after Bonferroni correction for multiple comparisons (*P* = 0.05/3≈0.017)

*Group A vs. Group C, *P* < 0.017;

^†^Group B vs. Group C, *P* < 0.017

^#^Group A vs. Group B, *P* < 0.017

### Comparison of chest CT features

**Fig. 3:**
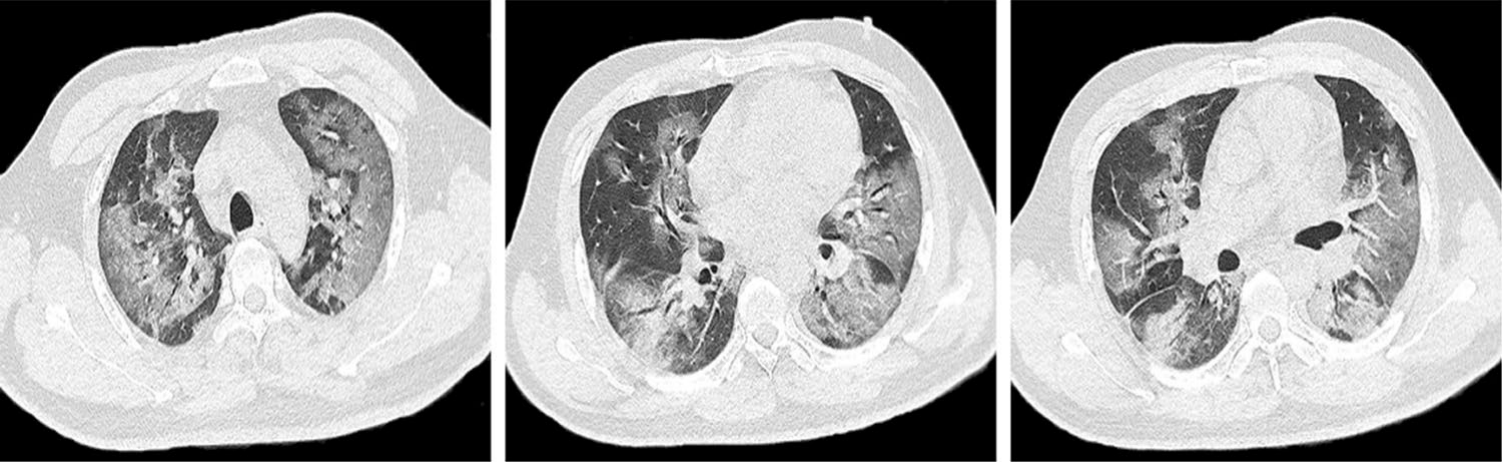
Chest CT feature the multiple lung segments involved. Female, 59 years, confirmed as COVID-19, chest CT images showed crazy-paving pattern and multiple lung segments involved

Table 2 reveals the chest CT features among Groups A, B, and C. Compared with the Group C, Groups A and B were more likely to shower crazy-paving pattern (24[92%] vs. 42[42%], 28[76%] vs. 42[42%], all *P* < 0.017). Group A and Group B (24[92%] vs. 28[76%], *P* > 0.017) were not significantly different. Furthermore, we found that the multiple (98%), irregular shape (93%), and GGO with consolidation (93%) were more common in the Groups A, B, and C. And the lesion-involved lung segment number in Groups A, B, and C were more than 15 (Fig. 3).

**Table 2:** Chest CT features among Groups A, B, and C

Features	Group A (*n* = 26)	Group B (*n* = 37)	Group C (*n* = 99)
Lesion numbers, No. (%)			
Single	0 (0)	1 (3)	3 (3)
Multiple	26 (100)	36 (97)	96 (97)
Lesion-involved lung segment number, No	16.2 ± 6.2	16.4 ± 5.4	15.6 ± 6.1
Lesion shape, No. (%)			
Round	7 (27)	15 (41)	38 (38)
Irregular shape	23 (88)	36 (97)	91 (92)
Lesion density, No. (%)			
GGO	3 (12)	1 (3)	5 (5)
Consolidation	1 (4)	0 (0)	0 (0)
GGO with consolidation	22 (85)	36 (97)	92 (93)
Crazy-paving pattern, No. (%)	24 (92)^*^	28 (76)^†^	42 (42)
Interstitial changes, No. (%)	20 (77)	31 (84)	81 (82)
Pleural effusion, No. (%)	2 (8)	4 (11)	4 (4)

*GGO* ground-glass opacity

*P* value after Bonferroni correction for multiple comparisons (*P* = 0.05/3≈0.017)

*Group A vs. Group C, *P* < 0.017

^†^ Group B vs. Group C, *P* < 0.017.

### Logistic regression analysis results

**Fig. 4:**
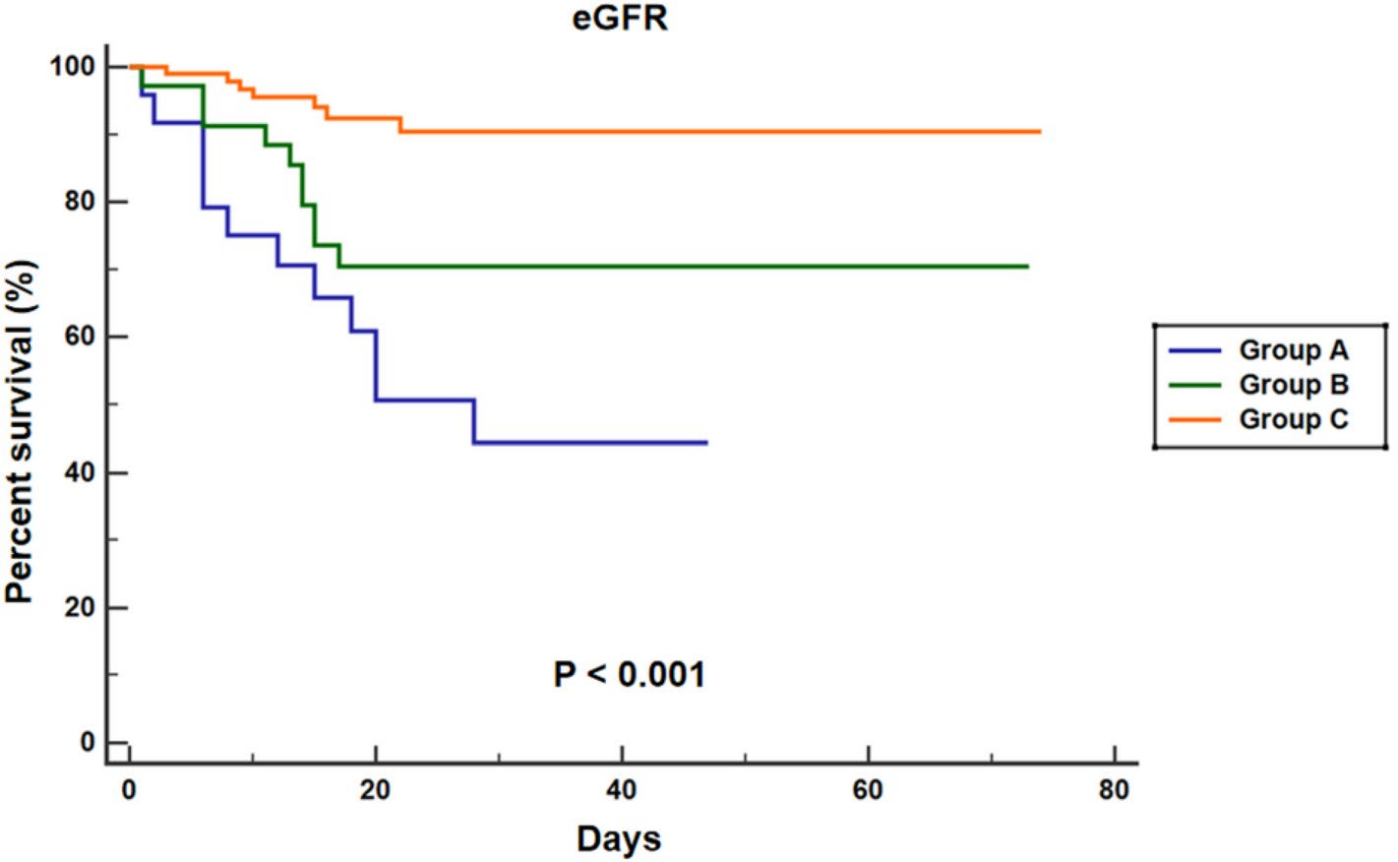
Kaplan–Meier curves for in-hospital mortality of patients with severe COVID-19 subgroup by eGFR

**Fig. 5:**
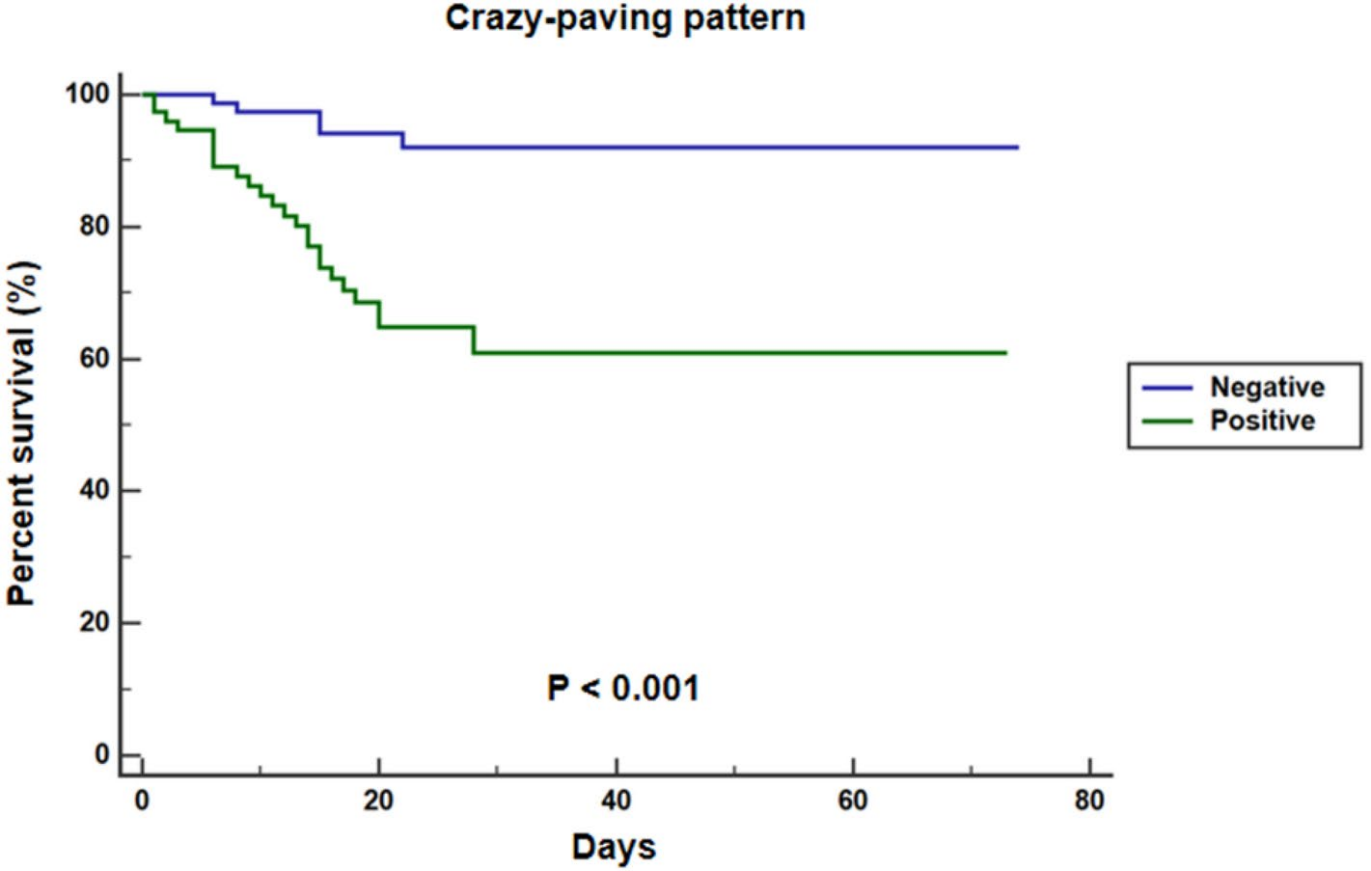
Kaplan–Meier curves for in-hospital mortality of patients with severe COVID-19 subgroup by Chest CT with or without crazy-paving pattern

Table 3 summarizes the risk factors related to crazy-paving pattern identified by logistic regression analysis. Univariate logistic regression analysis indicated that eGFR, PC, LDH were risk factors of crazy-paving pattern (all *P* < 0.05). The factors with *P* < 0.10 in univariate logistic regression analysis were selected to multivariate logistic regression analysis, which indicated that eGFR (OR = 0.962, 95% CI = 0.940–0.985, *P* = 0.001) was an independent risk factor of crazy-paving pattern. This shows that eGFR and crazy-paving pattern have a mutually reinforcing relationship. Univariate COX regression analysis indicated that age, eGFR, LC, and crazy-paving pattern were risk factors of mortality (all *P* < 0.05). The factors with *P* < 0.10 in univariate COX regression analysis were selected to multivariate COX regression analysis, which indicated that eGFR (HR = 0.549, 95% CI = 0.331–0.909, *P* = 0.020) and crazy-paving pattern (HR = 2.996, 95% CI = 1.010–8.714, *P* = 0.048) were independent risk factors of mortality (Table 4, Figs. 4, 5).

**Table 3:** Risk factors related to crazy-paving pattern identified by logistic regression analysis

	Univariate logistic regression		Multivariate logistic regression	
	OR (95%CI)	*P* value	OR (95%CI)	*P* value
eGFR	0.958 (0.942–0.974)	<0.001	0.962 (0.940–0.985)	0.001
Age	1.022 (1.000–1.044)	0.051	0.997 (0.954–0.985)	0.881
PC	0.991 (0.986–0.996)	0.001	0.994 (0.987–1.000)	0.060
CK	1.001 (1.000–1.002)	0.088	1.000 (0.999–1.001)	0.810
LDH	1.003 (1.000–1.005)	0.031	1.001 (0.998–1.004)	0.518
Lesion-involved lung segment number	1.049 (0.993–1.108)	0.085	0.962 (0.940–0.985)	0.332

*eGFR* estimated glomerular filtration rate, *PC* platelet count, *CK* creatine kinase, *LDH* Lactate dehydrogenase

**Table 4:** Risk factors related to mortality identified by COX regression analysis

	Univariate COX regression		Multivariate COX regression	
	HR(95%CI)	*P* value	HR(95%CI)	*P* value
Age	1.027 (1.001–1.053)	0.043	1.023 (0.995–1.053)	0.113
eGFR	0.379 (0.244–0.587)	<0.001	0.549 (0.331–0.909)	0.020
LC	0.271 (0.076–0.969)	0.045	0.369 (0.098–1.384)	0.139
Lesion-involved lung segment number	1.075 (0.994–1.163)	0.072	1.069 (0.987–1.159)	0.102
Crazy-paving pattern	5.924 (2.259–15.534)	<0.001	2.996 (1.010–8.714)	0.048

*eGFR* estimated glomerular filtration rate, *LC* Lymphocyte count

### Clinical prognosis

Table 5 demonstrated that the clinical prognosis of severe COVID-19 patients with or without crazy-paving pattern. The mortality of severe COVID-19 patients with crazy-paving pattern was significantly higher than that of patients without crazy-paving pattern (*P* < 0.05). However, there was no difference in the appearance of the crazy-paving pattern between those in enter ICU and mechanical ventilation adoption (all *P* > 0.05).

**Table 5:** The clinical prognosis of severe COVID-19 patients with crazy-paving pattern and without crazy-paving pattern

	Crazy-paving pattern (*n* = 94)	Non crazy-paving pattern (*n* = 68)	*P* value
Mortality, *n* (%)	32 (34)	13 (19)	0.032
The number of patients entering ICU, *n* (%)	49 (52)	26 (38)	0.080
Mechanical ventilation adoption, *n* (%)	45 (48)	29 (43)	0.444

## Discussion

With the wide outbreak of the COVID-19, it has been seriously affecting global public health. At present, the coronavirus has mutated and the WHO named it B.1617.2 (delta) variant. The B.1617.2 (delta) variant has become a major epidemic strain in the world [15]. Research confirmed that the B.1617.2 (delta) variant has the characteristics of strong infectivity and short incubation period, with the ability of easydevelopment into severe COVID-19 [16, 17]. In the present study, we have found that Group A (92%) and Group B (76%) are more likely to have crazy-paving pattern on chest CT than Group C (42%) which the severe COVID-19, with the appearance of the crazy-paving pattern on chest CT, are more likely to cause kidney injury (eGFR < 90 ml/min/1.73 m^2^).

Furthermore, we have found that when the patient’s serum creatinine value remains normal with the appearance of crazy-paving pattern on chest CT in severe COVID-19, doctors still need to calculate eGFR and monitor renal function. Relevant studies have shown that eGFR can found kidney injury earlier than serum creatinine [18]. According to the CKD clinical practice guidelines, eGFR less than 90 mL/min/1.73 m^2^ was considered as the occurence of kidney injury [19]. According to the guidelines for the diagnosis and treatment of COVID-19 (Trial Seventh Edition) issued by the National Health Commission of the People’s Republic of China, the chloroquine phosphate and ribavirin were listed as the first-line drugs for the treatment of COVID-19 [5]. However, the chloroquine phosphate and ribavirin have certain nephrotoxicity and can aggravate kidney injury [20, 21]. Meanwhile, the Chinese traditional medicine Qingfei Paidu Decoction was also listed as the first-line drugs for COVID-19 in guidelines, whereas the main ingredients of it was as arum, with nephrotoxicity as well [22]. Therefore, with the appearance of crazy-paving pattern on chest CT in severe COVID-19 suggesting possible kidney injury, doctors should consider reducing the dosage of chloroquine phosphate, ribavirin and Asarum or choose alternative drugs.

In our study, the mortality of severe COVID-19 with crazy-paving pattern on chest CT being significantly higher than that of patients without crazy-paving pattern (all *P* < 0.05) was further proved. In 1958, Rosen SH et al. first described crazy-paving pattern and proved that it can appear in pulmonary alveolar proteinosis (PAP) [23]. After that, crazy-paving pattern was also confirmed to be present in pneumocystis jirovecii pneumonia (PJP), cryptogenic organizing pneumonia (COP), sarcoidosis, bronchioloalveolar carcinoma, adult respiratory distress syndrome (ARDS), etc. [13, 24, 25]. Crazy-paving pattern might be associated with an interstitial inflammatory cellular infiltration or fibrosis [26]. Our study found more than 15 lung segments are involved and appeared crazy-paving pattern in Groups A, B, and C, which indicated the severity and numbers of lung involvement in severe COVID-19.

The reason for the kidney injury in severe COVID-19 still needs to be further explored. Relevant study reported that severe acute respiratory syndrome coronavirus 2 (SARS-CoV-2) may directly attack the tubular cells by binding angiotensin converting enzyme 2 (ACE2) receptor in the kidney, which may induce eGFR decline and kidney injury [27]. Moreover, COVID-19 mediated angiotensin II accumulation may promote renin–angiotensin–aldosterone (RAAS) activation, leading to inflammation, fibrosis and vasoconstriction. The eGFR decline might be secondary to inflammation, sepsis, shock or insufficient blood volume. Severe COVID-19 have elevated level of inflammatory cytokines, especially when they are admitted to the ICU. Last but not the least, the fibrin deposits in glomerular loops is in favor of a dysregulation of coagulation homeostasis that can participate in renal microcirculatory dysfunction and kidney injury [28].

This study has several limitations. Firstly, the sample size of this study is relatively small. Secondly, as the patients diagnosed with COVID-19 were immediately transferred to the designated isolation hospital, the dynamic results were missing. In the study, we adopt the quick baseline results of the first admission to the hospital. For the dynamic research results, we will further supplement and explore in the follow-up study. Thirdly, the values of eGFR was derived from the serum creatinine level, and was interfered with systemic conditions such as dehydration, fasting and the infection. There was still lack of robust and sensitive biomarkers of acute kidney injury. These problems will be further demonstrated in future study.

## Conclusions

The crazy-paving pattern is more likely to appear in the chest CT of patients with severe COVID-19. Among patients with severe COVID-19, the appearance of crazy-paving pattern in chest CT indicates the occurrence of kidney injury and proneness to death. The crazy-paving pattern can be adopted by doctors as an early warning indicator of kidney injury and clinical prognosis in severe COVID-19, and a guidance of reasonable drug selection.

## Data Availability

All data generated or analyzed during this study are included in this published paper.

## Abbreviations

ACE2: Angiotensin converting enzyme 2
AST: Aspartate aminotransferase
CK: Creatine kinase
COP: Cryptogenic organizing pneumonia
COVID-19: Coronavirus Disease 2019
CRP: C-reactive protein
eGFR: Estimated glomerular filtration rate
GGO: Ground-glass opacity
ICU: Intensive care unit
LC: Leukocyte count
LDH: Lactate dehydrogenase
PAP: Pulmonary alveolar proteinosis
PCP: Pneumocystis jirovecii pneumonia
PC: Platelet count
RAAS: Renin–angiotensin–aldosterone
ROC: Receiver operating characteristic
RR: Respiratory rate
SARS-CoV-2: Severe acute respiratory syndrome coronavirus 2
